# Cost-effectiveness analysis of a universal mass vaccination program with a PHiD-CV 2+1 schedule in Malaysia

**DOI:** 10.1186/s12962-017-0079-2

**Published:** 2017-08-22

**Authors:** Xiao Jun Wang, Ashwini Saha, Xu-Hao Zhang

**Affiliations:** 1 Department of Pharmacy, National University of Singapore, Block S4A, Level 3, 18 Science Drive 4, Singapore, 117543 Singapore; 2 GSK, 150 Beach Road, #22-00 Gateway West, Singapore, 189720 Singapore; 3 GSK Pharmaceutical Sdn Bhd, Level 6, Quill 9, 112 Jalan Semangat, 46300 Petaling Jaya, Selangor Malaysia

**Keywords:** Pneumococcal conjugate vaccines, Malaysia, Cost-effectiveness, PHiD-CV, PCV13

## Abstract

**Background:**

Currently, two pediatric pneumococcal conjugate vaccines are available in the private market of Malaysia—13-valent pneumococcal conjugate vaccine (PCV13) and pneumococcal polysaccharide and non-typeable *Haemophilus influenzae* protein D conjugate vaccine (PHiD-CV). This study aimed to evaluate the cost-effectiveness of a universal mass vaccination program with a PHiD-CV 2+1 schedule versus no vaccination or with a PCV13 2+1 schedule in Malaysia.

**Methods:**

A published Markov cohort model was adapted to evaluate the epidemiological and economic consequences of programs with no vaccination, a PHiD-CV 2+1 schedule or a PCV13 2+1 schedule over a 10-year time horizon. Disease cases, deaths, direct medical costs, quality-adjusted life-years (QALYs) and incremental cost-effectiveness ratios (ICERs) were estimated. Locally published epidemiology and cost data were used whenever possible. Vaccine effectiveness and disutility data were based on the best available published data. All data inputs and assumptions were validated by local clinical and health economics experts. Analyses were conducted from the perspective of the Malaysian government for a birth cohort of 508,774. Costs and QALYs were discounted at 3% per annum. One-way and probabilistic sensitivity analyses were performed.

**Results:**

Compared with no vaccination, a PHiD-CV 2+1 program was projected to prevent 1109 invasive pneumococcal disease (IPD), 24,679 pneumonia and 72,940 acute otitis media (AOM) cases and 103 IPD/pneumonia deaths over 10 years, with additional costs and QALYs of United States dollars (USD) 30.9 million and 1084 QALYs, respectively, at an ICER of USD 28,497/QALY. Compared with a PCV13 2+1 program, PHiD-CV 2+1 was projected to result in similar reductions in IPD cases (40 cases more) but significantly fewer AOM cases (30,001 cases less), with cost savings and additional QALYs gained of USD 5.2 million and 116 QALYs, respectively, demonstrating dominance over PCV13. Results were robust to variations in one-way and probabilistic sensitivity analyses.

**Conclusions:**

A PHiD-CV 2+1 universal mass vaccination program could substantially reduce pneumococcal disease burden versus no vaccination, and was expected to be cost-effective in Malaysia. A PHiD-CV 2+1 program was also expected to be a dominant choice over a PCV13 2+1 program in Malaysia.

**Electronic supplementary material:**

The online version of this article (doi:10.1186/s12962-017-0079-2) contains supplementary material, which is available to authorized users.

## Background

*Streptococcus pneumoniae* can result in a range of diseases, from invasive pneumococcal diseases (IPDs) (e.g. meningitis and bacteremia) to pneumonia and acute otitis media (AOM) [1]. Data from the World Health Organization (WHO) suggest that pneumococcal diseases caused around 480,000 deaths among children under 5 years of age in 2008 [2], making it the leading cause of vaccine-preventable death among children globally [3]. In addition, pneumonia has been reported as the fourth leading cause of death in 2009 among the Ministry of Health hospitals in Malaysia, accounting for 10.4% of inpatient deaths [4]. Although AOM is a much milder disease than pneumonia or IPDs, it is very common and is therefore associated with substantial impacts on healthcare costs and quality of life [5, 6]. Invasive disease, pneumonia and AOM can also be caused by microorganisms other than *S. pneumoniae*, including non-typeable *Haemophilus influenzae* (NTHi) [7, 8].

Pneumococcal conjugate vaccines (PCVs) were recommended as a priority for inclusion in national childhood immunization programs in all countries by the WHO in 2007 [9]. Although they are not currently included in the national immunization program in Malaysia, two PCVs are available in the private market of Malaysia: a 13-valent pneumococcal conjugate vaccine (PCV13; *Prevenar 13*) and a pneumococcal polysaccharide and NTHi protein D conjugate vaccine (PHiD-CV; *Synflorix*).

Two prior cost-effectiveness analyses have compared a 3+1 schedule using either PHiD-CV or PCV13 in Malaysia [10, 11], but the results are inconsistent. Aljunid et al. [10] predicted that PHiD-CV would be more cost-effective than PCV13, but Wu et al. [11] predicted the opposite. Unlike Aljunid et al. [10], Wu et al. [11] did not account for the protective effects of PHiD-CV in a number of key areas: (1) cross-protection against serotypes 6A and 19A, which has been demonstrated in a number of recent studies [12–14]; (2) indirect (herd) protection against IPD, which has been shown in surveillance data from Finland [15] and New Zealand [16]; and (3) protection against NTHi AOM, which has been shown in both the randomized controlled Clinical Otitis Media and Pneumonia Study (COMPAS) study [17] and the randomized controlled Pneumococcal Otitis Efficacy Trial (POET) study of PHiD-CV’s 11-valent precursor [18]. Wu et al.’s [11] methodology has recently been critiqued by Varghese et al. [19]. Excluding the protective effects of PHiD-CV in these key areas is against the current evidence and could have had a significant impact on the cost-effectiveness of PHiD-CV versus PCV13. It is therefore necessary to conduct another cost-effectiveness analysis, taking into account the latest evidence of vaccine effectiveness.

The goal of this economic evaluation was to assess the cost-effectiveness of universal mass pneumococcal vaccination with a PHiD-CV 2+1 vaccination strategy versus no vaccination or a PCV13 2+1 vaccination strategy from the perspective of the Malaysian government. The results from this study can provide scientific evidence for Malaysian healthcare policymakers to support their decision making on the introduction of PCV into the national immunization program.

## Methods

**Fig. 1:**
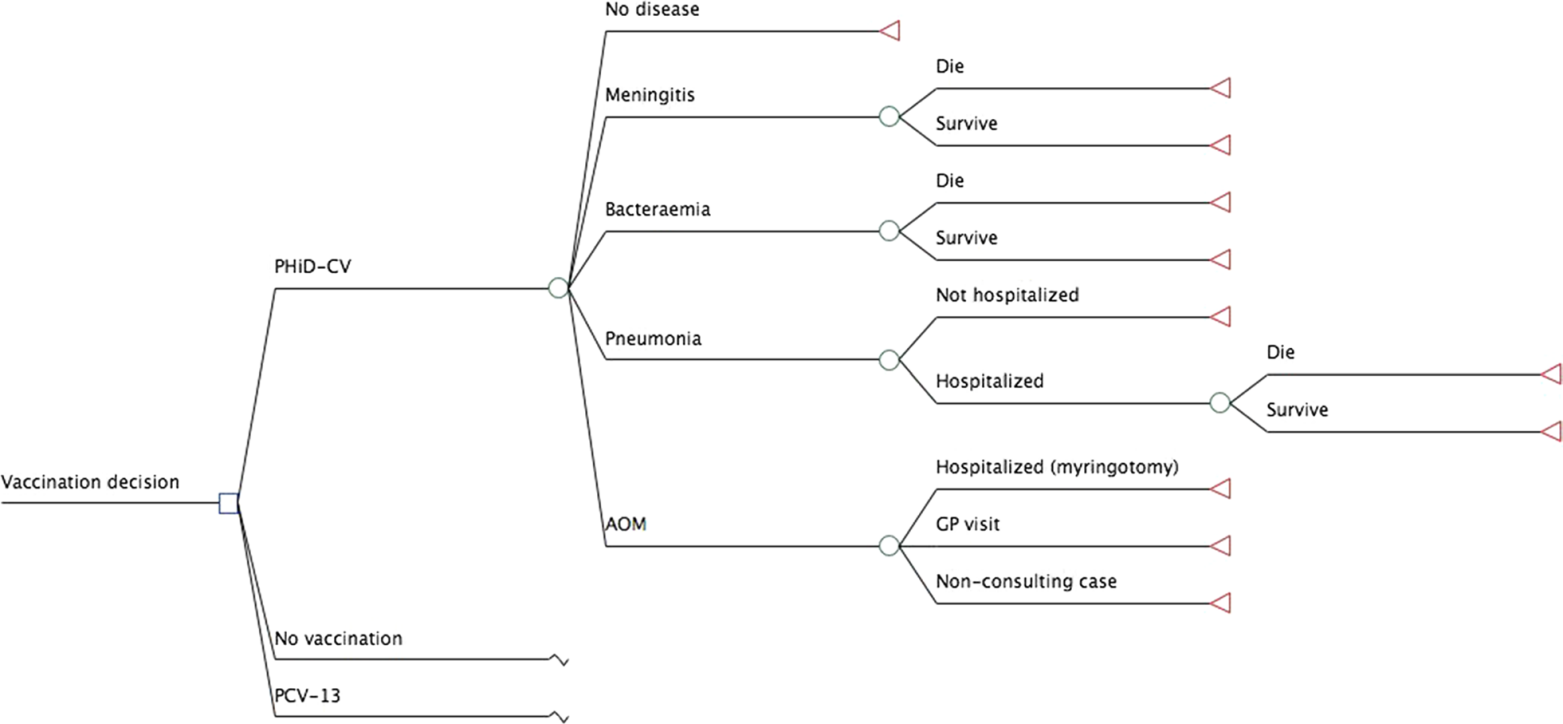
Model structure. *PHiD-CV* pneumococcal polysaccharide and NTHi protein D conjugate vaccine, *PCV13* 13-valent pneumococcal conjugate vaccine, *AOM* acute otitis media, *GP* general practitioner

A published Markov cohort model [20] was adapted to simulate the epidemiological and economic burden of pneumococcal and NTHi-related diseases (IPD, pneumonia and AOM) over a 10-year time horizon in Malaysia. In this study, infants could be vaccinated with PHiD-CV 2+1 or PCV13 2+1 or neither, as these are the vaccinations being considered for inclusion in the Malaysian universal mass vaccination program. The model has a number of mutually exclusive disease-related outcomes, namely pneumococcal meningitis, pneumococcal bacteremia, all-cause pneumonia, AOM and no pneumococcal infection (Fig. 1). Patients with AOM could be hospitalized (myringotomy), visit their general practitioner (GP) or be non-consulting. Patients with pneumonia could be treated as outpatients or inpatients. Those with meningitis, bacteremia or hospitalized pneumonia were at risk of death. Individuals of the birth cohort moved between the Markov states according to estimated transition probabilities. The reason for choosing 10 years’ time horizon in the base case is because we assumed that the duration of vaccine protection to be 10 years for the 2+1 regimen. In addition, it is because the serotypes are changing over time due to the vaccination program [21].

The analyses were conducted from the perspective of the Malaysian government for a birth cohort of 508,774 in 2012 [22]. Costs and quality-adjusted life-years (QALYs) were discounted at 3% per annum according to Malaysian guidelines [23]. All costs are reported in 2014 United States dollars (USD), converted when necessary from Malaysian Ringgits (MYR) using the exchange rate as at 31 Dec 2014 (3.5 MYR = 1 USD) [24]. The model and all-related data inputs and assumptions were reviewed and validated by a group of local clinical and health economics experts to ensure the validity of the model adaptation in the local setting.

### Epidemiological data (Table 1)

Age-specific incidence data for hospitalized pneumococcal meningitis and bacteremia (Additional file 1: Table S1) and hospitalized all-cause pneumonia (Additional file 1: Table S2) were sourced from a study that estimated disease burden from the hospital records of six tertiary hospitals across Malaysia during 2006–2007 [25]. Malaysian case fatality ratios (CFRs) were not available, so we referred to the CFRs from a retrospective population-based National Health Insurance Reimbursement Database study from Taiwan [26] (Additional file 1: Tables S1 and S2). This approach was conservative, considering Taiwan has a more advanced healthcare system and better healthcare access than Malaysia. GP consultation rates for all-cause pneumonia (Additional file 1: Table S2) were also lacking in Malaysia, but hospitalized pneumonia incidence rates were available. Therefore, an estimate was calculated, based on Malaysian hospitalization rates [25] and the ratio of Taiwanese GP consultation to hospitalization rates [26], considering similar healthcare access level within a country. Due to a lack of local/regional data on the long-term sequelae of IPD, these were not included.

**Table 1:** Country-specific model parameters for Malaysia

Parameter	Value in children aged <5 y	References	Range^a^	PSA distribution
Birth cohort size (2012)	508,774	[21]	Not varied	NA
Hospitalized pneumococcal meningitis				
Incidence (per 100,000)^b^	34.7	[25]	±50%	Triangular
CFR (%)^b^	12.5–19.2	[26]	±20%	Triangular
Hospitalized pneumococcal bacteremia				
Incidence (per 100,000)^b^	46.3	[25]	±50%	Triangular
CFR (%)^b^	4.1–4.9	[26]	±20%	Triangular
All-cause pneumonia				
GP consultation rate (per 100,000)^c^	4600–12,517	[25, 26]	±50%	Triangular
Hospitalization rate (per 100,000)^c^	765.8	[25]	±20%	Triangular
CFR (%)^c^	0.0–0.4	[26]	±20%	Triangular
All-cause AOM				
GP consultation rate (per 100,000)^d^	11,745–16,412	[27]	±50%	Triangular
Myringotomy procedures (per 100,000)^d^	10.8–27.2	[26, 27]	±20%	Triangular

*AOM* acute otitis media, *CFR* case fatality ratio, *GP* general practitioner, *PSA* probabilistic sensitivity analysis, *NA* not applicable

^a^Ranges used in the one-way sensitivity analyses

^b^Age-specific values are in Additional file 1: Table S1

^c^Age-specific values are in Additional file 1: Table S2

^d^Age-specific values are in Additional file 1: Table S3

Age-specific AOM GP consultation rates (Additional file 1: Table S3) were based on published data from the Philippines [27] due to the lack of Malaysian data. Myringotomy rates (Additional file 1: Table S3) were adapted from data from the Philippines [27] that were based on insurance data from Taiwan [26] and consultation with local ear, nose and throat experts. In the base case, all cases of AOM were assumed to result in a GP consultation. Complications and long-term sequelae of AOM were very conservatively not taken into account, due to a lack of suitable data.

### Vaccine effectiveness data (Table 2)

Vaccination was assumed to occur at ages 2, 4 and 13 months. In the base case, it was assumed that 100% of infants would be vaccinated, as WHO 2015 immunization coverage estimates for Malaysia for other childhood vaccines were close to 100% [28]. It was further assumed that all children would receive the defined number of doses at the recommended times, hence the effect of partial vaccination was not considered. Both vaccines were assumed to cost USD 34.25 per dose (hypothetical price). Due to a lack of local data, administration costs were not considered; and vaccine wastage was assumed to be 0%.

**Table 2:** Vaccine-specific model parameters

Parameter	PHiD-CV	PCV13	Range^a^	PSA distribution
Vaccination ages (months)	2, 4 and 13	2, 4 and 13	Not varied	NA
Vaccination coverage (%)	100 [28]	100 [28]	Not varied	NA
Vaccine cost (USD/dose)	34.25 (hypothetical price)	34.25 (hypothetical price)	Not varied	NA
VEs/effectivenesses (%)				
IPD				
Vaccine serotypes (excluding ST3)	94.7 (based on PCV7 data [31])	94.7 (based on PCV7 data [31])	95% CI^b^	Lognormal
ST3	0 (assumption)	26 (based on PCV13 data [32])	Not varied	NA
Cross-protection for ST6A	76.0 (based on PCV7 data [31, 36])	NA	95% CI^c^	Lognormal
Cross-protection for ST19A	82.2 (based on PHiD-CV surveillance [14])	NA	95% CI^c^	Lognormal
All-cause pneumonia				
Hospitalized	21.8 (based on PHiD-CV data [17])	21.8 (based on PHiD-CV data [17])	95% CI	Lognormal
GP visit	8.7 (based on PHiD-CV data [17])	8.7 (based on PHiD-CV data [17])	95% CI	Lognormal
All-cause AOM				
Vaccine serotypes (excluding ST3)	69.9 (based on PHiD-CV data [17])	69.9 (based on PHiD-CV data [17])	95% CI	Lognormal
Cross-protection for ST6A	63.7 (based on PHiD-CV precursor data [18])	NA	Not varied^c^	NA
Cross-protection for ST19A	61 (based on PHiD-CV and PCV7 data [14, 17, 31])^d^	NA	Not varied^c^	NA
ST3	0 (assumption)	0 (assumption due to a lack of relevant data)	Not varied	NA
Non-vaccine serotypes	–33 (based on PCV7 data [43])	–33 (based on PCV7 data [43])	95% CI	Lognormal
NTHi	21.5 (based on PHiD-CV data [17])	–11 (based on PCV7 data [43])	95% CI	Lognormal
Overall	24.1 (based on the above and pathogen split from [41, 42])	14.2 (based on the above and pathogen split from [41, 42])	Not varied	NA

*AOM* acute otitis media, *CI*, confidence interval, *GP* general practitioner, *IPD* invasive pneumococcal disease, *NA* not applicable, *NTHi* non-typeable *Haemophilus influenzae*, *PCV7* 7-valent pneumococcal conjugate vaccine, *PCV13* 13-valent pneumococcal conjugate vaccine, *PHiD-CV* pneumococcal polysaccharide and NTHi protein D conjugate vaccine, *ST* serotype, *USD* United States dollars, *VE* vaccine efficacy, *PSA* probabilistic sensitivity analysis

^a^Ranges used in the one-way sensitivity analyses

^b^Lowest and highest vaccine-type 95% CIs from [31] used for all serotypes

^c^Not applicable for PCV13

^d^Estimated based on PHiD-CV VE against 19A IPD [14], PCV7 VE against vaccine serotype IPD [31], and PHiD-CV VE against vaccine serotype AOM [17]

Vaccine effectiveness was assumed to have a ramp-up increase from age 2–13 months (50% after dose 1; 90% after dose 2), have full effectiveness from age 13 months to 3 years, and have waning effectiveness (exponential decline) to age 10 years [20].

Against IPD, PHiD-CV has been estimated to have vaccine efficacies (VEs) of 92% for the 2+1 schedule and 100% for the 3+1 schedule in the randomized, controlled Finnish Invasive Pneumococcal disease (FinIP) trial [29]. However, PCV13 has no published randomized controlled trial data against IPD. Therefore, vaccine effectiveness against IPD (pneumococcal meningitis and bacteremia) for both vaccines was estimated based on serotype distribution in Malaysia and serotype-specific efficacies. Serotype distribution was based on data from 217 invasive strains isolated in Malaysia in 2008–2009 [30] (Additional file 1: Figure S1). Based on the average VE of PCV7 against its seven serotypes [31], a VE of 94.7% was used for the 10 serotypes included in both vaccines (1, 4, 5, 6B, 7F, 9 V, 14, 18C, 19F, 23F) and for 6A and 19A for PCV13. However, serotype 3 in PCV13 has generally been shown to be less effective [32–35], so a VE of 26% was assumed [32]. Post-marketing surveillance studies of PHiD-CV have demonstrated cross-protection of PHiD-CV against 19A IPD with vaccine effectivenesses of 62% (95% confidence interval [CI] 20–85%) in Finland [13], 71% (95% CI 24–89%) in Canada [12] and 82.2% (95% CI 10.7–96.4%) in Brazil [14]. Based on local expert opinion, 82.2% from Brazil [14] was used, due to the potential similarity of economic status, healthcare systems and seasonality. Cross-protection of PHiD-CV against serotype 6A was based on cross-protection of PCV7 against serotype 6A (76.0% [31]), as PHiD-CV has been demonstrated to be immunogenically non-inferior to PCV7 [36].

Vaccine effectiveness against all-cause pneumonia is lower than against IPD as it can be caused by a number of pathogens. VE against all-cause pneumonia has been reported in various trials, with PCV7 (18% [95% CI 5–29%] [37]), PCV9 (20% [95% CI 2–35%] [38] and 35% [95% CI 26–43%] [39]), PHiD-CV (22% [95% CI 8–34%] [17]) and PCV11 (16% [95% CI –7 to 34%] [40]) (all intention-to-treat analyses), with no relationship between valency and VE. As there are no published randomized controlled studies for PCV13 against all-cause pneumonia, we used the value of 21.8% for consolidated pneumonia from COMPAS (PHiD-CV) [17] as the VE against pneumonia hospitalizations for both vaccines. VE against all-cause suspected pneumonia (8.7%) was taken from the same study [17] and was used for pneumonia associated with a GP visit.

Overall vaccine effectiveness against AOM was estimated based on causative pathogens (*S. pneumoniae*, *H. influenzae* or other) and VE against vaccine and non-vaccine *S. pneumoniae* serotypes and NTHi. Based on a review paper by Leibovitz et al. [41] of 17 AOM etiology studies across the world, we assumed that 35.9% of AOM cases were attributable to *S. pneumoniae* and 32.3% to NTHi. Data from a multinational AOM study were used for the percentages of AOM cases caused by each serotype [42]. VE against vaccine-type *S. pneumoniae* (excluding serotype 3) for both vaccines was taken to be 69.9% based on COMPAS [17]. For PHiD-CV, vaccine efficacies of 63.7% [18] and 61% [14, 17, 31] for serotypes 6A and 19A, respectively, were used. For both vaccines, a negative VE of −33% was used against other non-vaccine serotypes based on PCV7 data [43]. This study also gave a VE for PCV7 against *H. influenzae* AOM of −11% [43], which was used for PCV13. Two studies have demonstrated efficacy of PHiD-CV [17] (or its 11-valent precursor [18]) against NTHi AOM. The lower value of 21.5% (95% CI−43.4 to 57.0%) from COMPAS [17] was used. Although the 95% CI spans zero, it should be noted that the COMPAS trial [17] was not powered to provide conclusive evidence of protection against NTHi AOM. However, the positive effect of PHiD-CV against NTHi AOM is consistent with the significant efficacy observed with the 11-valent predecessor protein D conjugate formulation used in the POET study (35.3% [95% CI 1.8–57.4%]) [18]. The overall estimated vaccine effectivenesses against AOM were 24.1% for PHiD-CV and 14.2% for PCV-13.

No vaccine effectiveness for PHiD-CV against NTHi invasive disease or pneumonia was assumed due to a lack of evidence. Indirect effects (herd protection and serotype replacement) were also not taken into account in the base case.

### Health outcomes (Table 3)

Due to a lack of published pneumococcal disease-related disutility weights in Malaysia, short-term disutility data from the US [44] and Canada [45] were applied to age-specific healthy utilities [46].

**Table 3:** Short-term disutility weights of pneumococcal diseases

	Disutility weight	Reference/assumptions	Range^a^%	PSA distribution
Meningitis (inpatient)	0.023	[44] value for meningitis with recovery	±50	Beta (α = 7.70, β = 324.15)
Bacteremia (inpatient)	0.008	[44] value for hospitalization	±50	Beta (α = 6.46, β = 811.13)
Pneumonia (inpatient)	0.008	Assumed to be the same as for inpatient bacteremia	±50	Beta (α = 6.62, β = 821.25)
Pneumonia (outpatient)	0.006	[44] value for local infection	±50	Beta (α = 3.73, β = 618.18)
AOM (outpatient)	0.005	[45]	±50	Triangular
AOM (hospitalized myringotomy)	0.005	Assumed to be the same as for AOM (outpatient)	±50	Triangular

*AOM* acute otitis media, *PSA* probabilistic sensitivity analysis

^a^Ranges used in the one-way sensitivity analyses

### Treatment costs (Table 4)

Only direct medical costs were included. Direct medical costs for acute episodes were based on local published data from 2010 [10]. Costs were inflated using the consumer price index for heath for Malaysia [47] to 2014 values and then converted into USD [24].

**Table 4:** Costs for acute episodes of pneumococcal diseases [10]

	Weighted average cost (USD 2014)^a^	Range^b^ %	PSA distribution
Meningitis—hospitalized	1717	±20	Triangular
Bacteremia—hospitalized	838	±20	Triangular
Pneumonia—ospitalized	989	±20	Triangular
Pneumonia—outpatient	164	±20	Triangular
AOM—hospitalized (myringotomy)	583	±20	Triangular
AOM—GP consultation	191	±20	Triangular

*AOM* acute otitis media, *GP* general practitioner, *MYR* Malaysian Ringgits, *USD* United States dollars, *PSA* probabilistic sensitivity analysis

^a^2010 data in MYR from Aljunid et al. [10] (calculated as Cost all divided by Total cases per year) were inflated to 2014 values (consumer price index for heath for Malaysia of 111.4 [47]) and then converted to USD (3.5 MYR = 1 USD [24])

^b^Ranges used in the one-way sensitivity analyses

### Cost-effectiveness analysis

The model estimated cases, costs and QALYs specific to each health state over 10 years from birth for PHiD-CV 2+1, PCV13 2+1 or neither. Incremental cost-effectiveness ratios (ICERs) were computed for PHiD-CV 2+1 versus no vaccination and PHiD-CV 2+1 versus PCV13 2+1. Due to the lack of locally published threshold, the WHO threshold was used to categorize the cost-effectiveness results of this analysis [48]: a strategy was considered either as dominant (lower cost and more QALYs), highly cost-effective (ICER less than the gross domestic product [GDP] per capita of Malaysia [10,333 USD in 2014 [24, 49]]), cost-effective (ICER < 3 × GDP per capita [30,999 USD]) or not cost-effective (ICER ≥ 3 × GDP per capita).

### Sensitivity analyses

For comparisons of PHiD-CV 2+1 versus no vaccination or PCV13 2+1, extensive one-way sensitivity analyses were performed to evaluate the robustness of the results. These were mainly performed using values based on the upper and lower limits of 95% CIs for vaccine effectiveness and ±20 or ±50% of base-case values for most other parameters (see Tables 1, 2, 3, 4 for further details).

Probabilistic sensitivity analyses (PSAs) were performed for PHiD-CV 2+1 versus no vaccination or PCV13 2+1, each using 1000 simulations.

### Scenario analyses

A set of alternative scenario analyses on parameters of particular interest were performed for comparisons of PHiD-CV 2+1 versus no vaccination or PCV13 2+1: (1) discount rates of 0 and 5, as per Malaysian guidelines [23]; (2) a lower proportion of AOM cases due to NTHi (20% rather than 32.3%); (3) a different adjustment factor for estimating total AOM cases (i.e. consulting plus non-consulting cases) (0.7 and 1.3 rather than 1.0); (4) a time horizon of 100 rather than 10 years; (5) inclusion of indirect effects (herd protection and serotype replacement) for all age groups and both vaccines (only applied to IPD [50]); it was assumed that this would reach a steady state of 30% (i.e. 30% reduction in disease incidence).

## Results

### Cost-effectiveness analysis

#### PHiD-CV 2+1 versus no vaccination

It was projected that vaccination with a PHiD-CV 2+1 program would prevent 1109 cases of IPD, 24,679 cases of all-cause pneumonia, 72,940 cases of AOM and 103 IPD/pneumonia deaths compared with no vaccination strategy for the birth cohort of 508,774 in Malaysia over 10 years (Table 5).

**Table 5:** Estimated disease burden impacts of no vaccination, PCV13 2+1 and PHiD-CV 2+1 vaccination programs

	No vaccination	PCV13	PHiD-CV	PHiD-CV versus no vaccination	PHiD-CV versus PCV13
IPD cases (acute episodes)	2444	1295	1335	–1109	+40
Meningitis	1021	533	550	–471	+17
Bacteremia	1423	762	785	–638	+23
All-cause pneumonia cases (acute episodes)	534,819	510,143	510,140	–24,679	–3
AOM cases (acute episodes)	565,764	522,825	492,824	–72,940	–30,001
Pneumococcal deaths	239	132	136	–103	+4
IPD	209	107	111	–98	+4
Pneumonia	30	25	25	–5	0

*AOM* acute otitis media, *IPD* invasive pneumococcal disease, *PCV13* 13-valent pneumococcal conjugate vaccine, *PHiD-CV* pneumococcal polysaccharide and NTHi protein D conjugate vaccine

In Malaysia over 10 years for a birth cohort of 508,774

Undiscounted vaccination costs were estimated to be USD 52.1 million, but these would be partially offset by a reduction in direct medical costs of USD 22.4 million, for a total direct medical cost of USD 29.7 million (Table 6). Discounted total cost and QALY increases were predicted to be USD 30.9 million and 1084 QALYs, respectively, demonstrating a cost-effective ICER of USD 28,497/QALY.

**Table 6:** Estimated economic impacts of no vaccination, PCV13 2+1 and PHiD-CV 2+1 vaccination programs

	No vaccination	PCV13	PHiD-CV	PHiD-CV versus no vaccination	PHiD-CV versus PCV13
Undiscounted costs (USD)					
Vaccination costs	0	52,119,711	52,119,681	+52,119,681	–30
Acute episode costs					
IPD	2945,307	1,553,212	1,602,129	–1,343,178	+48,917
Meningitis	1,753,066	914,936	944,383	–808,683	+29,447
Bacteremia	1,192,241	638,276	657,746	–534,495	+19,470
All-cause pneumonia	106,306,148	99,249,563	99,249,006	–7,057,142	–557
AOM	108,394,044	100,136,093	94,366,242	–14,027,802	–5,769,851
Total direct costs	217,645,499	253,058,579	247,337,058	+29,691,559	–5,721,521
QALYs	4,590,144	4,591,267	4,591,392	+1,248	+125
ICER				23,792	PHiD-CV dominant
Discounted costs (USD)^a^					
Total direct costs	190,226,159	226,321,520	221,112,241	+30,886,082	–5,209,279
QALYs	3,979,331	3,980,299	3,980,415	+1084	+116
ICER	–	–	–	28,497 (cost-effective)	PHiD-CV dominant

Costs are in 2014 USD

*AOM* acute otitis media, *ICER* incremental cost-effectiveness ratio, *IPD* invasive pneumococcal disease, *PCV13* 13-valent pneumococcal conjugate vaccine, *PHiD-CV* pneumococcal polysaccharide and NTHi protein D conjugate vaccine, *QALY* quality-adjusted life-year, *USD* United States dollars

In Malaysia over 10 years for a birth cohort of 508,774

^a^Discounted at 3% per annum [23]

#### PHiD-CV 2+1 versus PCV13 2+1

It was projected that vaccination with a PHiD-CV 2+1 program would result in 40 more IPD cases than vaccination with a PCV13 2+1 program, but 30,001 fewer cases of AOM (Table 5). Similarly, undiscounted direct medical costs with PHiD-CV 2+1 were predicted to be somewhat higher for IPD, but substantially lower for AOM, resulting in an overall cost saving of USD 5.7 million (Table 6). Discounted total cost savings and QALYs gained were predicted to be 5.2 million and 116, respectively, meaning that PHiD-CV 2+1 was predicted to be dominant over PCV13 2+1 (lower cost and more QALYs gained).

### Sensitivity analyses

#### PHiD-CV 2+1 versus no vaccination

**Fig. 2:**
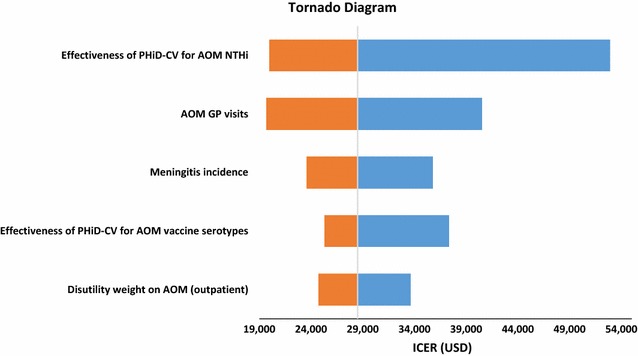
Top five most influential factors identified in one-way sensitivity analysis for the cost-effectiveness of PHiD-CV 2+1 versus no vaccination. *PHiD-CV* pneumococcal polysaccharide and NTHi protein D conjugate vaccine, *AOM* acute otitis media, *NTHi* non-typeable *Haemophilus influenzae, GP* general practitioner, *USD* United States dollars

**Fig. 3:**
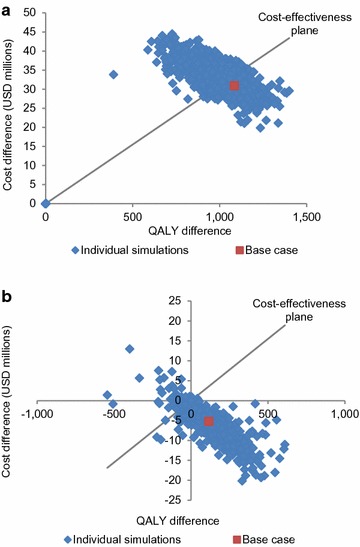
PSA results for PHiD-CV 2+1 versus **a** no vaccination and **b** PCV13 2+1 vaccination programs. The “cost-effective” threshold was taken to be 3 × GDP per capita. *GDP* gross domestic product, *PCV13* 13-valent pneumococcal conjugate vaccine, *PHiD-CV* pneumococcal polysaccharide and NTHi protein D conjugate vaccine, *PSA* probabilistic sensitivity analysis, *QALY* quality-adjusted life-year, *USD* United States dollars

According to one-way sensitivity analyses, the most influential factors on the cost-effectiveness of PHiD-CV 2+1 versus no vaccination were the efficacy of PHiD-CV against NTHi AOM, the AOM GP consultation rate, and the incidence of hospitalized pneumococcal meningitis (Fig. 2; Additional file 1: Table S4). In the PSA, the probability of PHiD-CV being cost-effective compared with no vaccination was 31.6% at the cost-effectiveness threshold for Malaysia (Fig. 3a).

#### PHiD-CV 2+1 versus PCV13 2+1

**Fig. 4:**
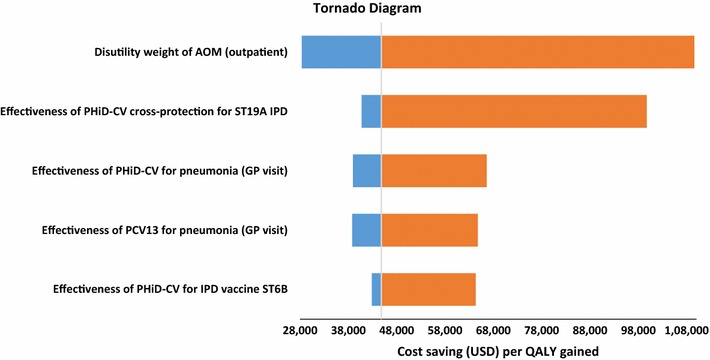
Top five most influential factors identified in one-way sensitivity analysis for the cost-effectiveness of PHiD-CV 2+1 versus PCV13 2+1. *AOM* acute otitis media, *PHiD-CV* pneumococcal polysaccharide and NTHi protein D conjugate vaccine, *ST19A* serotype 19A, *IPD* invasive pneumococcal disease, *GP* general practitioner, *PCV13* 13-valent pneumococcal conjugate vaccine, *ST6B* serotype 6B, *USD* United States dollars

One-way sensitivity analyses showed that the most influential factors were the disutility weight for AOM (outpatient) and PHiD-CV cross-protection for serotype 19A IPD (Fig. 4); but none of the varied model inputs impacted on the dominant conclusion of PHiD-CV 2+1 over PCV13 2+1 (Additional file 1: Table S4). The PSA showed PHiD-CV 2+1 to be dominant over PCV13 2+1 in 91.6% of the simulations (Fig. 3b).

### Scenario analyses

Most of the scenario analyses predicted that PHiD-CV 2+1 would be cost-effective versus no vaccination; running the model over 100 years resulted in a prediction that PHiD-CV 2+1 would be highly cost-effective (Table 7). Scenario analyses predicted PHiD-CV 2+1 to be dominant over PCV13 2+1 for all scenarios tested.

**Table 7:** Scenario analyses of PHiD-CV 2+1 versus no vaccination or PCV13 2+1 vaccination programs in Malaysia

Parameter	Base case	Scenario analysis	PHiD-CV versus no vaccination (ICER)	PHiD-CV versus PCV13 (cost saving [USD million]/QALYs gained)
Base case	–	–	28,497^a^	5.2/116
Discount rate	3%	0%	23,792^a^	5.7/125
Discount rate	3%	5%	31,793	4.9/110
AOM cases due to NTHi	32.3%	20.0%	30,843^a^	3.1/60
Adjustment factor for total AOM cases	1.0	0.7	31,371	5.2/75
Adjustment factor for total AOM cases	1.0	1.3	26,105^a^	5.2/157
Time horizon	10 years	100 years	10,317^b^	5.2/50
Indirect effects	Excluded	Included	21,032^a^	5.2/116

*AOM* acute otitis media, *ICER* incremental cost-effectiveness ratio, *NTHi* non-typeable *H. influenzae*, *PCV13* 13-valent pneumococcal conjugate vaccine, *PHiD-CV* pneumococcal polysaccharide and NTHi protein D conjugate vaccine, *QALY* quality-adjusted life-year, *USD* United States dollars

^a^Cost-effective (<USD 30,999)

^b^Highly cost-effective (<USD 10,333)

## Discussion

In this cost-effectiveness analysis, the adoption of pediatric mass vaccination with a PHiD-CV 2+1 program was predicted to prevent 1109 cases of IPD, 24,679 cases of pneumonia, 72,940 cases of AOM, and 103 IPD/pneumonia-related deaths over 10 years compared with no vaccination program in Malaysia. PHiD-CV 2+1 was predicted to result in 1084 QALYs gained at a cost of USD 30.9 million (discounted), resulting in a cost-effective ICER of USD 28,497/QALY. Our results are in line with various other health economic studies that have predicted that the introduction of routine infant vaccination with PHiD-CV would be cost-effective, including those in Georgia [51], Latin America [52–55] and Kenya [56].

Compared with a PCV13 2+1 program, a PHiD-CV 2+1 program was predicted to result in 40 more IPD cases, but 30,001 fewer cases of AOM. This was expected to result in more QALYs gained at a lower overall cost, i.e. PHiD-CV 2+1 was predicted to be dominant over PCV13 2+1. This dominance is in line with various other cost-effectiveness analyses, from Malaysia [10], Japan [57], the Philippines [27], Turkey [58], Europe [20, 59–61], Canada [20] and Peru [53]. However, some studies have predicted the reverse: that PCV13 would be dominant over PHiD-CV (Malaysia and Hong Kong [11], Colombia [62], Europe [63] and Canada [64]) or more cost-effective than PHiD-CV (Peru [65]). These differences in predicted outcomes are largely due to the assumptions used. As noted in a recently published critical assessment of economic evaluations involving PHiD-CV and PCV13, “the pivotal assumptions and results of these analyses also depended on which manufacturer sponsored the study” [66].

We will focus our discussion on the key differences in assumptions of a prior Malaysian study by Wu et al. [11] (PCV13 dominance) compared with those used in the current study (PHiD-CV dominance): (1) inclusion of herd effects for PCV13 but not PHiD-CV; (2) no cross-protection for PHiD-CV against serotypes 6A and 19A; (3) higher effectiveness of PCV13 against all-cause pneumonia; and 4) no impact of PHiD-CV on NTHi AOM. In addition, recent evidences has shown lower VE for PCV13 against serotype 3 IPD [32–35], which was not reflected in the previous study by Wu et al. [11].

In the current study, we conservatively chose not to include herd effect for PHiD-CV or PCV13 in the base case, as this would be counteracted by serotype replacement, resulting in an inconclusive overall effect. In one of the scenario analyses, we included an overall beneficial indirect effect. The same net value was used for PHiD-CV and PCV13, as expert advice was that both herd effect and serotype replacement would likely be slightly higher for PCV13, giving a similar net effect. However, it should be noted that inclusion of indirect effects for PCV13, but not for PHiD-CV is considered as unrealistic, given that a population-based observational study of PHiD-CV in Finland [13] reported a 48% (95% CI 18–69%) reduction in IPD among unvaccinated children aged 2–5 years. Additionally, surveillance data have demonstrated herd effects of PHiD-CV following the introduction of childhood vaccination programs in Finland [15] and New Zealand [16].

In the current study, cross-protection of PHiD-CV against non-vaccine serotypes 6A and 19A was accounted for, while no cross-protection was assumed for PHiD-CV against non-vaccine serotypes in the prior Malaysian study [11]. For serotype 19A, this was based on a number of robustly designed studies [12–14]. The available evidences have recently prompted the European Medicines Agency to include protection against 19A IPD in PHiD-CV’s label [67]. A similar label update was approved in Canada [68] and is underway in many countries. Although the evidence for cross-protection against serotype 6A is less conclusive, the data generally indicate that cross-protection is likely [13, 14] and would be similar to that seen with PCV7 [43, 69].

We assumed that PHiD-CV and PCV13 would have equal effectivenesses against all-cause pneumonia, based on a number of studies of different valent vaccines with overlapping 95% CIs [17, 37–40]. It is inappropriate to assume that vaccine effectiveness against all-cause pneumonia would be related to the number of serotypes in the vaccine. This type of serotype-based approach has recently been criticized by Hausdorff et al. [70], because currently available pneumonia efficacy data provide no indication of a trend for greater protection with higher valence vaccines.

Lastly, cautions should be made when assuming that PHiD-CV had no effect against NTHi AOM, merely based on a few studies in which PHiD-CV was shown to have no effect on nasopharyngeal colonization [71–73] without looking into other available evidences. Several randomized controlled studies have reported a beneficial effect of PHiD-CV (or its 11-valent precursor) against NTHi AOM [17, 18]; and Prymula et al. [18] also reported a 41.4% (95% CI −4.9 to 67.3%) VE against nasopharyngeal carriage. Furthermore, Australian studies have reported less NTHi-infected middle-ear discharge among those with AOM with perforation or chronic suppurative otitis media vaccinated with PHiD-CV versus PCV7 (35% vs.53% of ear discharge swabs; p = 0.03) [74] and with PHiD-CV versus PCV13 (36% vs 64% of swabs; p = 0.05) [75]. Also, a randomized controlled PCV7 trial [43] showed that the number of *H. influenzae* AOM cases was increased in the PCV7 group. In the absence of PCV13-specific data, we assumed that PCV13 would also increase NTHi AOM cases (vaccine efficacy −11%).

Guidelines from the International Society for Pharmacoeconomics and Outcomes Research recommend that all evidence—not selected sources—should be incorporated into health economic studies [76]. By incorporating the wider body of evidence available around these critical parameters and employing robust sensitivity analyses, we believe that we have addressed the associated uncertainties and attempted to present a more balanced result. As mentioned previously, outcomes of health economic studies of PHiD-CV and PCV13 can be biased, depending on the sponsor [66]. Therefore, decision makers should be encouraged to rigorously evaluate the underlying assumptions in all cost-effectiveness analyses.

### Strengths and limitations

As discussed above, the results of cost-effectiveness analyses very much depend on the input parameters used. In the absence of a head-to-head PHiD-CV versus PCV13 trial, we based effectiveness estimates on serotype distribution and data from various clinical trials. Additionally, there are also no randomized controlled PCV13 efficacy studies, hence we extrapolated effectiveness (based on serotype distribution and PCV7 vaccine serotype VE) or assumed values to be the same as for PHiD-CV. We note that VE data were taken from studies of 3+1 schedules, whereas the current model used a 2+1 schedule. This approach was based on the results of a PHiD-CV trial that reported similar vaccine effectivenesses of 100% (95% CI 83–100%) for a 3+1 schedule and 92% (95% CI 58–100%) for a 2+1 schedule against vaccine-type IPD [29]. Therefore, it is likely that we slightly overestimated the effectiveness of a PHiD-CV 2+1 schedule. However, the same approach was taken for PCV13, so this would have had little effect on the between-vaccine comparison.

Although Malaysian data were used where possible, this was not always feasible, so some data from other countries (Asian when available) had to be used; but this was approved by local clinical experts. However, for some inputs, it was not possible to find any suitable sources, so administration costs, other vaccine program-related costs (e.g. capital costs, logistics costs) and vaccine wastage were all assumed to be zero. Although this could overestimate the cost-effectiveness of PHiD-CV versus no vaccination, there would be no impact on the between-vaccine comparison. We also assumed that all children would receive the recommended number of doses, but in reality, it is likely that some children would not receive the full schedule. This would have resulted in a slightly lower efficacy but also slightly lower costs.

Lastly, the cost-effectiveness thresholds that we used were those recommended by the WHO at the time of the study [48]. These have recently been criticized [77], but in the absence of new recommendations, we have used the old thresholds.

## Conclusions

In this cost-effectiveness analysis, a PHiD-CV 2+1 universal vaccination program could potentially prevent a substantial number of cases of pneumococcal diseases compared with no vaccination, and was projected to be a cost-effective strategy in Malaysia. A PHiD-CV 2+1 vaccination program was also predicted to be dominant (more QALYs gained at a reduced overall cost) over a PCV13 2+1 strategy.

## Additional file

**Additional file 1.** Additional input data and one-way sensitivity analysis results.

## Abbreviations

AOM: acute otitis media
CFR: case fatality ratio
CI: confidence interval
COMPAS: Clinical Otitis Media and Pneumonia Study
FinIP: Finnish Invasive Pneumococcal disease
GDP: gross domestic product
GP: general practitioner
ICER: incremental cost-effectiveness ratio
IPD: invasive pneumococcal disease
MYR: Malaysia Ringgits
NTHi: non-typeable *Haemophilus influenzae*
PCV: pneumococcal conjugate vaccine
PCV13: 13-valent pneumococcal conjugate vaccine
PHiD-CV: pneumococcal polysaccharide and NTHi protein D conjugate vaccine
POET: Pneumococcal Otitis Efficacy Trial
PSA: probabilistic sensitivity analysis
QALY: quality-adjusted life-year
USD: United States dollars
VE: vaccine efficacy
WHO: World Health Organization
