# Supplementary material for: Cost-effectiveness analysis of a universal mass vaccination program with a PHiD-CV 2+1 schedule in Malaysia
Source: Cost Eff Resour Alloc. 2017 Aug 22;15:17. doi: 10.1186/s12962-017-0079-2 (PMC5568314; doi:10.1186/s12962-017-0079-2)
Supplement: Supplementary file 1 — Additional file 1. Additional input data and one-way sensitivity analysis results. [file 12962_2017_79_MOESM1_ESM.docx]

**Additional file**

**Table S1 Incidence and CFR data for hospitalized pneumococcal meningitis and bacteremia**

| Age group (years)^a^ | Pneumococcal meningitis | | Pneumococcal bacteremia | |
| --- | --- | --- | --- | --- |
|  | Incidence (per 100,000) [25] | CFR (%) [26] | Incidence (per 100,000) [25] | CFR (%) [26] |
| <1 | 34.7 | 19.2 | 46.3 | 4.9 |
| 1 | 34.7 | 19.2 | 46.3 | 4.9 |
| 2–4 | 34.7 | 12.5 | 46.3 | 4.1 |
| 5–9 | 5.7 | 12.5 | 10.0 | 2.5 |
| 10–19 | 5.7 | 6.4 | 10.0 | 11.7 |
| 20–39 | 3.5 | 6.4 | 3.5^b^ | 11.7 |
| 40–64 | 2.7 | 6.4 | 2.7^b^ | 11.7 |
| 65–69 | 4.6 | 6.4 | 4.6^b^ | 11.7 |
| ≥70 | 4.6 | 6.3 | 4.6^b^ | 11.6 |

^a^Age-specific information beyond age 10 years old was used in a life-time analysis (alternative scenario 4).

^b^Incidence data for pneumococcal bacteremia from Aljunid et al. [25] among people aged ≥20 years were felt to be too high (based on local expert opinion); therefore, the same incidences as for pneumococcal meningitis were used.

CFR, case fatality ratio.

**Table S2 GP consultation rates, hospitalization rates, and CFR data for all-cause pneumonia**

| Age group (years)^a^ | GP consultation rate (per 100,000) [25,26] | Hospitalization rate (per 100,000) [25] | CFR (%) [26] |
| --- | --- | --- | --- |
| <1 | 4,600 | 765.8 | 0.4 |
| 1 | 6,317 | 765.8 | 0.1 |
| 2 | 8,049 | 765.8 | 0.1 |
| 3 | 9,305 | 765.8 | 0.1 |
| 4 | 12,517 | 765.8 | 0.0 |
| 5–9 | 12,137 | 126.1 | 0.1 |
| 10–14 | 14,832 | 126.1 | 0.4 |
| 15–19 | 13,804 | 126.1 | 1.4 |
| 20–24 | 9,163 | 93.3 | 1.0 |
| 25–29 | 17,129 | 93.3 | 2.1 |
| 30–34 | 18,188 | 93.3 | 3.2 |
| 35–39 | 16,780 | 93.3 | 4.7 |
| 40–44 | 48,135 | 352.5 | 6.6 |
| 45–49 | 33,668 | 352.5 | 7.5 |
| 50–54 | 24,486 | 352.5 | 7.2 |
| 55–59 | 13,356 | 352.5 | 7.2 |
| 60–64 | 9,550 | 352.5 | 6.6 |
| 65–69 | 37,795 | 2,040.6 | 6.7 |
| 70–74 | 26,423 | 2,040.6 | 8.2 |
| 75–79 | 17,656 | 2,040.6 | 9.9 |
| 80–84 | 8,327 | 2,040.6 | 11.2 |
| 85–89 | 3,014 | 2,040.6 | 12.3 |
| ≥90 | 1,148 | 2,040.6 | 13.7 |

^a^Age-specific information beyond age 10 years old was used in a life-time analysis (alternative scenario 4).

CFR, case fatality ratio; GP, general practitioner.

**Table S3 GP consultation and myringotomy procedure rates for all-cause AOM**

| Age group (years)^a^ | GP consultation rate (per 100,000) [27] | Myringotomy procedures (per 100,000)^b^ [26,27] |
| --- | --- | --- |
| <1 | 16,412 | 10.8 |
| 1 | 16,412 | 20.7 |
| 2 | 11,745 | 16.6 |
| 3 | 11,745 | 20.6 |
| 4 | 11,745 | 27.2 |
| 5–9 | 8,751 | 14.5 |
| 10–14 | 1,496 | 4.3 |
| 15–19 | 1,496 | 1.7 |
| 20–24 | 1,496 | 2.3 |
| 25–29 | 1,496 | 2.3 |
| 30–34 | 1,496 | 3.0 |
| 35–39 | 1,496 | 3.9 |
| 40–44 | 1,496 | 4.3 |
| 45–49 | 1,496 | 4.3 |
| 50–54 | 1,496 | 5.6 |
| 55–59 | 1,496 | 8.8 |
| 60–64 | 1,496 | 9.3 |
| 65–69 | 1,508 | 10.8 |
| 70–74 | 1,508 | 11.9 |
| 75–79 | 1,508 | 10.2 |
| 80–84 | 1,508 | 8.0 |
| 85–89 | 1,508 | 5.8 |
| ≥90 | 1,508 | 3.8 |

^a^Age-specific information beyond age 10 years old was used in a life-time analysis (alternative scenario 4).

^b^Based on data from the Philippines [27] that were adapted based on insurance data from Taiwan [26]; and consultation with local ear, nose and throat specialists.

AOM, acute otitis media; GP, general practitioner.

**Table S4 Results of the one-way sensitivity analyses**

| Parameters varied | PHiD-CV vs. no vaccination  (cost per QALY gained) | | | PHiD-CV vs. PCV13  (cost per QALY gained)^a^ | | |
| --- | --- | --- | --- | --- | --- | --- |
|  | Lower bound | Higher bound | Difference | Lower bound | Higher bound | Difference |
| Epidemiological parameters |  |  |  |  |  |  |
| Meningitis incidence | 35,766 | 23,589 | 12,177 | –42,461 | –48,109 | 5,648 |
| Meningitis CFRs | 30,847 | 26,513 | 4,334 | –43,925 | –46,233 | 2,308 |
| Bacteremia incidence | 31,040 | 26,340 | 4,700 | –44,021 | –46,132 | 2,111 |
| Bacteremia CFRs | 29,359 | 27,720 | 1,639 | –44,614 | –45,494 | 880 |
| Pneumonia GP visits | 31,567 | 25,766 | 5,801 | –45,049 | –45,051 | 2 |
| Pneumonia hospitalizations | 29,447 | 27,605 | 1,842 | –45,050 | –45,050 | 0 |
| Pneumonia CFRs | 28,679 | 28,355 | 324 | –45,050 | –45,050 | 0 |
| AOM GP visits | 40,532 | 19,682 | 20,850 | –42,511 | –54,854 | 12,343 |
| AOM hospitalized myringotomies | 28,546 | 28,487 | 59 | –44,993 | –45,106 | 113 |
| Vaccine effectiveness |  |  |  |  |  |  |
| PHiD-CV for IPD vaccine serotypes^b^ |  |  |  |  |  |  |
| ST1 | 29,200 | 28,377 | 823 | –43,526 | –56,508 | 12,982 |
| ST4 | 28,605 | 28,478 | 127 | –44,805 | –46,527 | 1,722 |
| ST5 | 28,642 | 28,472 | 170 | –44,723 | –47,040 | 2,317 |
| ST6B | 29,543 | 28,321 | 1,222 | –42,842 | –64,353 | 21,511 |
| ST7F | 28,533 | 28,490 | 43 | –44,970 | –45,533 | 563 |
| ST9V | 28,497 | 28,497 | 0 | –45,053 | –45,053 | 0 |
| ST14 | 29,275 | 28,364 | 911 | –43,372 | –58,075 | 14,703 |
| ST18C | 28,863 | 28,434 | 429 | –44,236 | –50,405 | 6,169 |
| ST19F | 29,238 | 28,371 | 867 | –43,449 | –57,280 | 13,831 |
| ST23F | 28,863 | 28,434 | 429 | –44,236 | –50,405 | 6,169 |
| PCV13 for IPD vaccine serotypes^b^ |  |  |  |  |  |  |
| ST1 | NA | NA | NA | –37,583 | –46,697 | 9,114 |
| ST4 | NA | NA | NA | –43,674 | –45,304 | 1,630 |
| ST5 | NA | NA | NA | –43,234 | –45,388 | 2,154 |
| ST6A | NA | NA | NA | –40,769 | –45,903 | 5,134 |
| ST6B | NA | NA | NA | –34,888 | –47,518 | 12,630 |
| ST7F | NA | NA | NA | –44,583 | –45,136 | 553 |
| ST9V | NA | NA | NA | –45,053 | –45,053 | 0 |
| ST14 | NA | NA | NA | –36,946 | –46,877 | 9,931 |
| ST18C | NA | NA | NA | –40,769 | –45,903 | 5,134 |
| ST19A | NA | NA | NA | –36,946 | –46,877 | 9,931 |
| ST19F | NA | NA | NA | –37,262 | –46,787 | 9,525 |
| ST23F | NA | NA | NA | –40,769 | –45,903 | 5,134 |
| PHiD-CV cross-protection for ST6A IPD | 28,954 | 28,327 | 627 | –42,921 | –51,931 | 9,010 |
| PHiD-CV cross-protection for ST19A IPD | 30,441 | 28,138 | 2,303 | –40,754 | –99,734 | 58,980 |
| PHiD-CV for pneumonia (hospitalized) | 31,584 | 26,052 | 5,532 | –38,947 | –47,790 | 8,843 |
| PHiD-CV for pneumonia (GP visit) | 31,969 | 25,528 | 6,441 | –38,942 | –66,611 | 27,669 |
| PCV13 for pneumonia (hospitalized) | NA | NA | NA | –40,258 | –48,172 | 7,914 |
| PCV13 for pneumonia (GP visit) | NA | NA | NA | –38,774 | –64,753 | 25,979 |
| PHiD-CV for AOM vaccine serotypes | 37,346 | 25,300 | 12,046 | –31,796 | –42,842 | 11,046 |
| PHiD-CV for AOM non-vaccine serotypes | 31,160 | 26,694 | 4,466 | –43,695 | –48,738 | 5,043 |
| PHiD-CV for AOM NTHi | 52,885 | 19,964 | 32,921 | –34,074 | –41,244 | 7,170 |
| PCV13 for AOM vaccine serotypes^b^ | NA | NA | NA | –41,651 | –51,113 | 9,462 |
| PCV13 for AOM non-vaccine serotypes | NA | NA | NA | –43,387 | –47,146 | 3,759 |
| PCV13 for AOM NTHi | NA | NA | NA | –42,214 | –63,031 | 20,817 |
| Disutility weights |  |  |  |  |  |  |
| Meningitis (inpatient) | 28,632 | 28,368 | 264 | –44,981 | –45,123 | 142 |
| Bacteremia (inpatient) | 28,560 | 28,436 | 124 | –45,019 | –45,086 | 67 |
| Pneumonia (inpatient) | 28,858 | 28,146 | 712 | –45,053 | –45,053 | 0 |
| Pneumonia (outpatient) | 30,053 | 27,094 | 2,959 | –45,050 | –45,055 | 5 |
| AOM (outpatient) | 33,633 | 24,721 | 8,912 | –28,357 | –109,556 | 81,199 |
| AOM (hospitalized; myringotomy) | 28,511 | 28,482 | 29 | –44,964 | –45,141 | 177 |
| Costs |  |  |  |  |  |  |
| Meningitis (hospitalized) | 28,516 | 28,516 | 0 | –45,050 | –45,050 | 0 |
| Bacteremia (hospitalized) | 28,607 | 28,425 | 182 | –45,019 | –45,081 | 62 |
| Pneumonia (hospitalized) | 29,134 | 27,899 | 1,235 | –45,050 | –45,050 | 0 |
| Pneumonia (outpatient) | 29,082 | 27,950 | 1,132 | –45,049 | –45,050 | 1 |
| AOM (hospitalized; myringotomy) | 28,540 | 28,492 | 48 | –44,958 | –45,141 | 183 |
| AOM (GP consultation) | 30,848 | 26,184 | 4,664 | –36,054 | –54,045 | 17,991 |

Costs are in 2014 USD.

^a^Negative cost per QALY gained values represent cost savings.

^b^Excluding ST3.

AOM, acute otitis media; CFR, case fatality ratio; GP, general practitioner; IPD, invasive pneumococcal disease; NTHi, non-typeable *Haemophilus influenzae*; PCV13, 13-valent pneumococcal conjugate vaccine; PHiD-CV, pneumococcal polysaccharide and NTHi protein D conjugate vaccine; QALY, quality-adjusted life-year; ST, serotype; USD, United States dollars

**Figure S1** Invasive strain serotype distribution in Malaysia (217 isolates) in 2008–2009 [30].

**References**

Reference numbers match those in the reference list in the main manuscript.
